# Efficacy and safety of apalutamide, abiraterone acetate, and bicalutamide in the treatment of metastatic hormone-sensitive prostate cancer

**DOI:** 10.3389/fonc.2025.1656216

**Published:** 2025-10-13

**Authors:** Jiabin Zhang, Qiang Wang, Junjie Zhou, Huiyu Gao, Peng Hao, Tao Wu

**Affiliations:** ^1^ Department of Urology, Affiliated Hospital of North Sichuan Medical College, North Sichuan Medical College, Nanchong, China; ^2^ Department of Urology, Deyang Hospital Affiliated to Chengdu University of Traditional Chinese Medicine, Deyang, China; ^3^ Department of Urology, Dazhou Dachuan District People's Hospital China (Dazhou Third People’s Hospital), Dazhou, China

**Keywords:** apalutamide, abiraterone acetate, bicalutamide, prostate cancer, endocrine therapy, androgen deprivation therapy

## Abstract

**Objectives:**

To compare the efficacy and safety of three drugs—Apalutamide, Abiraterone, and Bicalutamide—combined with Androgen Deprivation Therapy (ADT) in patients with metastatic hormone-sensitive prostate cancer (mHSPC).

**Methods:**

We retrospectively collected survival data of patients treated at our hospital from January 2019 to March 2024. Patients who received three different treatment regimens—Apalutamide (240 mg/day) combined with ADT, Abiraterone (1000 mg/day) plus Prednisone (5 mg/day) combined with ADT, and Bicalutamide (50 mg/day) combined with ADT.

**Results:**

This study analyzed 146 mHSPC patients. The results are displayed that Apalutamide and Abiraterone significantly prolonged PFS and PSA-PFS compared to Bicalutamide. Univariate and multivariate COX regression analyses suggested that factors such as age <75 years, absence of lymph node metastasis, use of Apalutamide or Abiraterone, and a low ECOG score were associated with longer PFS. Moreover, Apalutamide and Abiraterone showed superior efficacy in improving PSA response compared to Bicalutamide. Importantly, no life-threatening adverse events were reported in any of the three treatment groups.

**Conclusion:**

Compared to Bicalutamide, the novel endocrine therapies Apalutamide and Abiraterone both significantly prolong PFS, PSA-PFS, and improve PSA response rates.

## 1 Introduction

Metastatic hormone-sensitive prostate cancer (mHSPC) refers to prostate cancer (PCa) that responds to endocrine therapy and is associated with bone or other organ metastases. In 1941, Huggins and colleagues first discovered the androgen dependency of PCa and proposed androgen deprivation therapy (ADT), which focuses on blocking androgen signaling as a core treatment strategy for PCa (1). Currently, androgen deprivation therapy (ADT) is widely regarded as the cornerstone of systemic therapy for mHSPC (2). ADT involves reducing serum testosterone levels to ≤50 ng/dl (castration level) through pharmacological or surgical methods, thereby diminishing the effects of androgens in the serum to block androgen action (3). ADT combined with endocrine therapy has been proven to effectively slow tumor growth, thereby improving patients’ survival outcomes and quality of life. Early ADT treatment can provide good disease control, but over time, the diminishing therapeutic effect is common, leading to the metastatic castration-resistant prostate cancer (mCRPC) stage.

Early-stage prostate cancer often lacks obvious clinical symptoms, and in China, the vast majority of patients are already in the middle or late stages at diagnosis (4). Subgroup analysis of the TITAN trial in East Asia also showed that, compared to Western populations, the incidence of prostate cancer is lower in East Asian countries, but East Asian populations tend to present at more advanced stages at initial diagnosis (5). Maximizing the time from mHSPC progression to mCRPC and improving patient survival and prognosis are the current focal points of discussion among clinicians. In recent years, in addition to traditional non-steroidal anti-androgen drugs (e.g., bicalutamide), the emergence of new endocrine drugs (e.g., abiraterone acetate, apalutamide, darolutamide, enzalutamide) has significantly transformed the treatment landscape of mHSPC.

Abiraterone acetate is a novel endocrine drug that selectively inhibits the cytochrome P450 isoform 17 (CYP17). By inhibiting CYP17A1, abiraterone effectively reduces androgen levels in the body. In recent years, with the successive results of large randomized controlled trials (RCTs) such as LATITUDE and STAMPEDE (6–8), studies have shown that abiraterone significantly prolongs the progression-free survival (PFS) and overall survival (OS) of mHSPC patients. Apalutamide, like bicalutamide, is an androgen receptor (AR) antagonist, but it has a stronger binding affinity for AR, inhibiting its activity and suppressing the growth and spread of prostate cancer cells. Recent high-quality studies on apalutamide have demonstrated that, in patients with metastatic hormone-sensitive prostate cancer, apalutamide combined with standard first-line therapy can significantly prolong PFS and OS (5, 9, 10). Japanese researchers have also reported studies comparing the efficacy and prognosis of abiraterone versus bicalutamide and apalutamide versus bicalutamide in high-risk mHSPC patients. The results consistently show that both abiraterone and apalutamide provide advantages in extending progression-free survival over bicalutamide, to varying degrees (11–14).

We conducted this retrospective study to compare the efficacy and adverse events of apalutamide, abiraterone, and bicalutamide in mHSPC patients. This study aims to provide valuable reference for clinicians and offer insights into further exploration of the treatment effects of novel endocrine therapies for mHSPC.

## 2 Materials and methods

**Figure 1:**
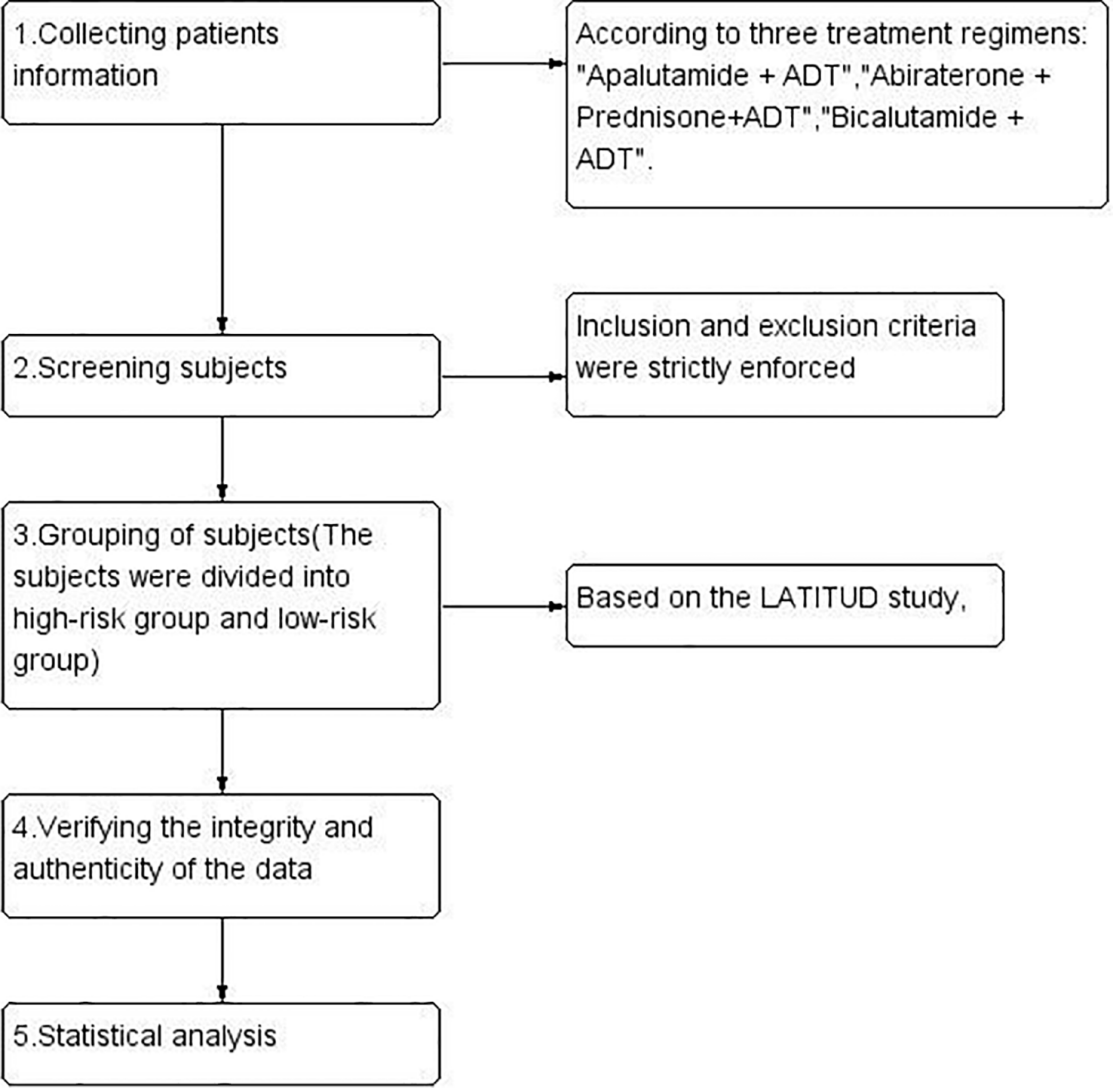
Study design and operational workflow.

We retrospectively collected data from our hospital between January 2019 and March 2024 on patients with metastatic hormone-sensitive prostate cancer (mHSPC) who received apalutamide (240 mg/day) combined with ADT (Group A), abiraterone (1000 mg/day) plus prednisone (5 mg/day) combined with ADT (Group B), or bicalutamide (50 mg/day) combined with ADT (Group C). All patients were required to continue using ADT (except for those who underwent bilateral orchiectomy), with ADT mainly involving LHRH agonists (such as goserelin acetate, triptorelin acetate, leuprorelin acetate) to maintain serum testosterone levels below 50 ng/dL (<1.7 nmol/L). The study design and flowchart are referenced in (**Figure 1**).

After strictly applying the inclusion and exclusion criteria (**Supplementary File 1**), we ultimately screened 146 patients. Based on the risk stratification from the LATITUDE study (6), patients were categorized into high-risk and low-risk disease groups for subgroup analysis (hereafter referred to as the high-risk/low-risk groups). This study aims to focus on disease progression(PSA progression/Imaging progression) (15, 16), PSA response at three months after treatment (nPSA: the lowest serum PSA level reached by patients after endocrine therapy, defined in this study as <0.2 ng/mL; PSA50: PSA decline ≥50%; PSA90: PSA decline ≥90%), and the incidence of adverse events. The effective follow-up period is from the initiation of treatment until disease progression or death; once disease progression or death occurs, follow-up ends. Adverse events were assessed using the Common Terminology Criteria for Adverse Events (CTCAE) version 5.0. The study protocol was approved by the Ethics Committee of the Affiliated Hospital of North Sichuan Medical College (Approval No: 2024ER130-1). In accordance with the Declaration of Helsinki, this study is classified as a retrospective study and the institutional review board waived the requirement for individual written informed consent.

We used IBM SPSS Statistics 26 software for statistical analysis and finished survival curves. Kaplan-Meier survival curves were used to assess differences in PFS among the three treatment regimens, and Cox univariate and multivariate regression analyses were performed to identify factors affecting PFS. The forest diagram was drawn in R (version 4.3.0) software. The chi-square test and Wilcoxon rank-sum test were appropriately used to compare the three groups. All results were considered statistically significant at P<0.05.

## 3 Results

A total of 146 patients were included in this retrospective study and there were no significant statistical differences in the baseline characteristics of the patients(patient characteristics are provided in the **Supplementary File 2: Table 1**); 42 patients in Group A, 57 in Group B, and 47 in Group C. We conducted a statistical analysis of the observed parameters in these patients. Both the treatment regimens in Group A and Group B significantly improved disease progression, PSA progression, and PSA response compared to Group C, with no significant differences observed between Group A and Group B. Additionally, the incidence of radiographic progression was lower in Groups A and B compared to Group C, though the difference was not statistically significant (**Table 1**).

**Table 1:** Compares disease progression and PSA response among the three patient groups.

Observational parameters	A (n=42)	B (n=57)	C (n=47)	p_value
Disease progression (Yes, n)	9(21.4%)	14(24.6%)	29(61.7)	<0.001
PSA response (Yes, n)				
nPSA	25(59.5%)	33(57.9%)	15(31.9%)	0.011
PSA90	29(69.0%)	40(70.1%)	16(34.0%)	<0.001
PSA50	36(85.7%)	47(82.5%)	25(53.2%)	<0.001
PSA progression (Yes, n)	7(16.7%)	13(22.8%)	27(57.4%)	<0.001
Radiographic progression (Yes, n)	4(9.5%)	6(10.5%)	12(25.5%)	0.051

**Figure 2:**
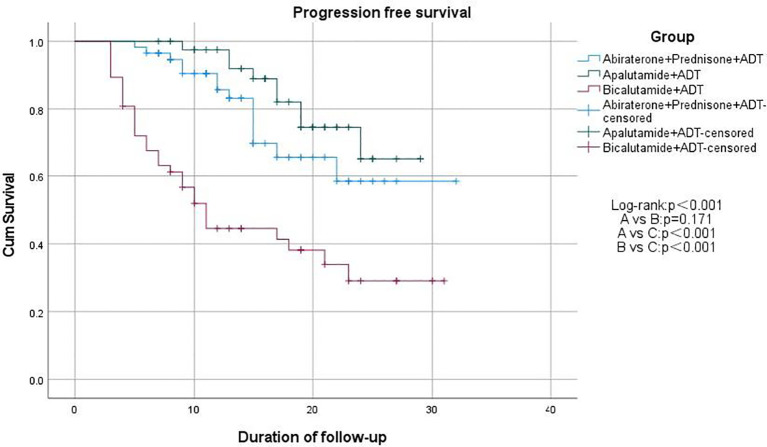
Kaplan–Meier estimates of progression free survival.

**Figure 3:**
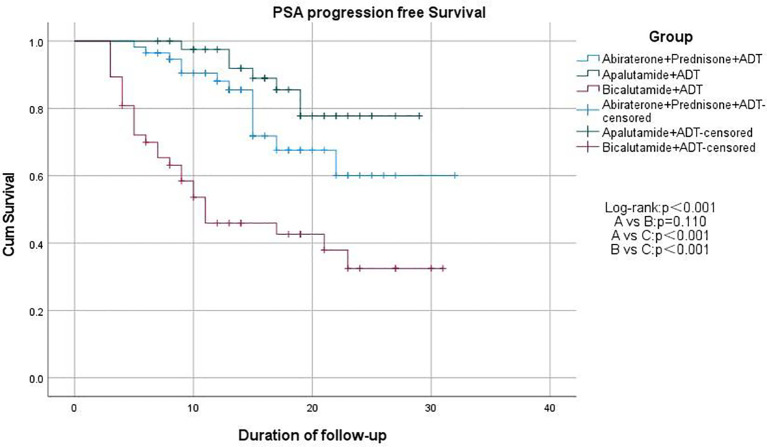
Kaplan–Meier estimates of PSA progression free survival.

We used Kaplan-Meier survival curves to evaluate patients’ progression-free survival (PFS) and performed statistical analysis using the log-rank test. We found that treatment regimens A and B significantly prolonged PFS compared to regimen C (p < 0.001), but there was no significant difference between regimens A and B (p = 0.171; **Figure 2**). Additionally, we compared PSA-PFS among the three groups, and the results were similar to those for PFS (**Figure 3**). The median PFS and PSA-PFS in Group C were both 11 months, while neither was reached in Groups A and B.

**Figure 4:**
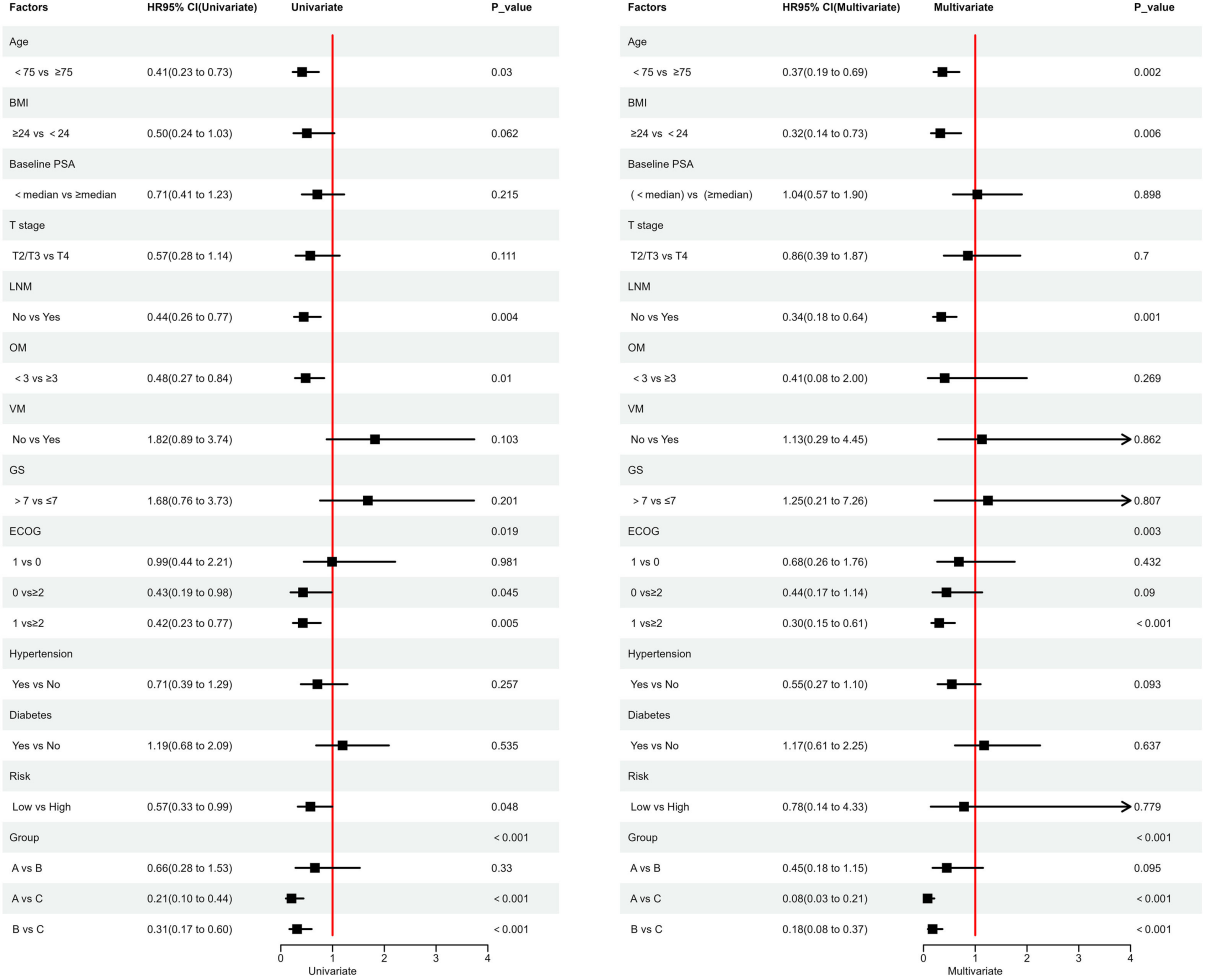
Cox Univariate and Multivariate Regression Analysis; ECOG: Represents the Eastern Cooperative Oncology Group Performance Status.

Finally, we used Cox univariate and multivariate regression analyses to explore the impact of various factors on PFS. Regression analysis revealed that factors such as age < 75 years, absence of lymph node metastasis, treatment with regimens A or B, and ECOG performance status were associated with longer PFS. Notably, patients with an ECOG performance status score of 1 had significantly longer PFS compared to those with a score of ≥2 (**Figure 4**).

### 3.1 Subgroup analysis

We conducted subgroup analyses by stratifying patients into high-risk and low-risk groups based on the risk stratification from the LATITUDE study. In the high-risk group, the median PFS for Group C was 7 months, and the median PSA-PFS was 8 months, while in the low-risk group, both the median PFS and PSA-PFS for Group C were 11 months. Neither Group A nor Group B reached the median PFS or PSA-PFS, and the differences were statistically significant. In the high-risk group, we also found that patients in Groups A and B had significantly better outcomes in terms of disease progression, PSA response, and PSA progression compared to Group C. The incidence of radiographic progression in Groups A and B was lower than that in Group C; however, the difference was not statistically significant. In the low-risk group, both Groups A and B significantly alleviated disease progression and PSA progression compared to Group C, achieving an increase in PSA90. Although Groups A and B were superior to Group C in nPSA, PSA50, and radiographic progression, these differences were not statistically significant (**Supplementary File 2: Tables 2, 3**). We also employed Kaplan-Meier survival curves to evaluate PFS and PSA-PFS for both groups, using the log-rank test for statistical analysis. We found that treatment regimens A and B significantly prolonged both PFS and PSA-PFS compared to regimen C, with no significant difference between regimens A and B (**Supplementary File**: **Figures 1**-**4**).

### 3.2 Adverse events

During the treatment with the three drugs, we summarized the types and frequencies of adverse events experienced by patients, as well as those classified as grade ≥3 (**Supplementary File: Table 4**). The main adverse events in Group A included pain, fatigue, and hot flashes. Group B primarily reported edema, pain, and fatigue. Group C mainly included pain, gastrointestinal reactions, and sleep disturbances. No life-threatening serious adverse events occurred with any of the three drugs. We conducted a summary analysis of the results (**Table 2**); the incidence of adverse events in Group A was 59.5%, which was higher than that in Group B (45.6%) and Group C (48.9%), but the difference was not statistically significant (p = 0.376). The incidence of grade ≥3 adverse events in Group A (19.0%) was slightly higher than that in Group B (17.5%) and Group C (10.6%), but overall, the results also showed no statistical differences (p = 0.495).

**Table 2:** Comparison of adverse event incidence among the three patient groups.

Frequencies of adverse events	A(n=42)	B(n=57)	C(n=47)	p_value
Total	25(59.5%)	26(45.6%)	23(48.9%)	0.376
Grade ≥3	8(19.0%)	10(17.5%)	5(10.6%)	0.495

## 4 Discussion

In this retrospective study, we found that apalutamide and abiraterone significantly prolonged both PFS and PSA-PFS, with consistent results observed in the subgroup analyses. According to Cox regression analysis, factors such as age < 75 years, absence of lymph node metastasis, treatment with apalutamide or abiraterone, and ECOG performance status (patients with a score of 1 showed significantly longer PFS compared to those with a score ≥2) were associated with prolonged PFS. Both apalutamide and abiraterone were significantly more effective than bicalutamide in improving disease progression, PSA progression, and PSA response. The incidence of radiographic progression was lower for both drugs compared to bicalutamide, although this difference was not statistically significant. Similar results were observed in the high-risk group. In the low-risk group, patients treated with apalutamide and abiraterone showed better nPSA and PSA50 outcomes compared to bicalutamide, though the differences were not statistically significant. Other indicators followed similar trends as previously described. In terms of adverse events, the incidence was higher with apalutamide than with abiraterone, and slightly higher with abiraterone compared to bicalutamide. The incidence of grade ≥3 adverse events was similar across all three groups, with no statistically significant differences.

Currently, androgen receptor axis-targeted agent (ARAT) therapy has become a standard treatment for patients with mHSPC. Several studies comparing apalutamide with bicalutamide, and abiraterone with bicalutamide, have shown that apalutamide combined with ADT is superior to bicalutamide in terms of overall survival (OS) and PSA-PFS. Compared to bicalutamide, abiraterone acetate can prolong the time to mCRPC in high-risk mHSPC patients. Additionally, abiraterone significantly reduces the incidence of castration resistance compared to bicalutamide (50.6% vs. 25.2%, P<0.001) (12–14, 17). These findings are consistent with the results of our study. However, in those studies, the dose of bicalutamide was 80 mg/day, while in our study, the dose was 50 mg/day. The difference in dosage may have influenced the therapeutic benefits observed in patients. Overall, the emergence of new endocrine agents such as apalutamide and abiraterone has demonstrated significantly superior clinical benefits compared to bicalutamide.

In our study, we found that patients aged >75 years were at higher risk for shorter PFS, possibly due to the increased risk of being diagnosed with Gleason score ≥7 cancer as age increases, which may affect PFS (18). However, our study found no significant association between Gleason score (>7 vs ≤7) and PFS, which may be attributed to the small sample size. On the other hand, related studies have shown that lymph node metastasis is a prognostic factor for recurrence-free survival, metastasis-free survival, and overall survival in prostate cancer patients (19), which may partially explain our findings. Additionally, our study found that a higher ECOG performance status was a risk factor for shorter PFS. This may be partly because a higher proportion of patients with ECOG score ≥2 in our data were over 75 years old. Older patients tend to have a higher likelihood of being diagnosed with high-risk disease and exhibit lower tolerance to tumors.

Regarding PSA response, Boegemann M et al.’s latest study demonstrated that in mHSPC patients treated with apalutamide combined with ADT, 94.4% achieved PSA50, 70.8% achieved PSA90, and 42.2% achieved uPSA (uPSA is defined similarly to nPSA in this article) after 3 months of treatment. These findings are consistent with the results of this study. Additionally, both Boegemann M and Encarnación Navarro JA highlighted in their published research that in subgroup analyses of M1a metastatic patients, apalutamide combined with ADT still provided significant clinical benefits (20, 21). This discovery supplements the findings of the TITAN study. The study by Benjamin Lowentritt, M.D., et al. found that at 6 months of follow-up, a higher proportion of patients receiving apalutamide achieved PSA90 compared to those receiving abiraterone (66.2% vs. 43.4%) (22). By 9 months, the proportion of patients in the apalutamide group who achieved PSA90 remained higher (68.1% vs. 47.4%). The results for apalutamide in that study are similar to our findings. However, the results for abiraterone acetate differ somewhat from our data. The results from Benjamin Lowentritt, M.D., et al.’s study are significantly lower than those of our study. Additionally, our study reports a slightly lower PSA90 response compared to the LATITUDE *post-hoc* analysis (79.3% at the end of follow-up) (23). We hypothesize that this may be due to some patients not adhering to follow-up schedules and the relatively small sample size. In addition, some current studies have proposed that the implementation of metastasis-directed therapy (MDT) for mHSPC patients through stereotactic body radiation therapy (SBRT) combined with androgen receptor signaling inhibitors (ARSI) can significantly improve the PSA response rate. This strategy helps delay further systemic treatment for patients and may also postpone the onset of castration resistance (24, 25).

There are several limitations to this study, primarily that it is a retrospective analysis, and all participants were drawn from a single institution, which may introduce selection bias. Additionally, the follow-up period was not long enough to fully reveal overall survival differences. Furthermore, the generalizability of the findings may be limited due to the small sample size. Based on these factors, we hope that future studies with larger sample sizes and longer follow-up durations will be conducted to further validate these preliminary findings.

## 5 Conclusions

In patients with mHSPC, both novel endocrine agents apalutamide and abiraterone were shown to prolong PFS and PSA-PFS compared to bicalutamide. Apalutamide and abiraterone also reduced the risk of disease progression and improved PSA response rates, with no life-threatening or rare adverse events observed.

## Data Availability

The raw data supporting the conclusions of this article will be made available by the authors, without undue reservation.
