# Supplementary material for: Efficacy and safety of apalutamide, abiraterone acetate, and bicalutamide in the treatment of metastatic hormone-sensitive prostate cancer
Source: Front Oncol. 2025 Oct 13;15:1656216. doi: 10.3389/fonc.2025.1656216 (PMC12554570; doi:10.3389/fonc.2025.1656216)
Supplement: Supplementary file 1 [file DataSheet1.docx]

**Supporting Information**

Table 1. Baseline Characteristics of Patients.

Table 2. Comparison of Disease Progression and PSA Response Among the Three Groups of High-Risk Patients.

Table 3. Comparison of Disease Progression and PSA Response Among the Three Groups of Low-Risk Patients.

Figure 1: Kaplan-Meier Survival Curve for PFS in the High-Risk Group.

Figure 2: Kaplan-Meier Survival Curve for PFS in the Low-Risk Group.

Figure 3: Kaplan-Meier Survival Curve for PSA-PFS in the High-Risk Group.

Figure 4: Kaplan-Meier Survival Curve for PSA-PFS in the Low-Risk Group.

Table 4: Occurrence of Adverse Events in the Three Patient Groups.

**Table 1**: Baseline Characteristics of Patients.

|  | A(n=42) | B(n=57) | C(n=47) | p_value |
| --- | --- | --- | --- | --- |
| Age | 71.9±7.9 | 75(69.5-78) | 75.0±6.2 | 0.291 |
| ＜75 | 25 | 26 | 21 |  |
| ≥75 | 17 | 31 | 26 |  |
| BMI（kg/m²） |  |  |  | 0.856 |
| ＜24 | 31 | 43 | 37 |  |
| ≥24 | 11 | 14 | 10 |  |
| Initial PSA | 141.6(45.6-312.7) | 108.6(35.0-202.9) | 103.2(23.8-154.8) | 0.266 |
| T Stage |  |  |  | 0.920 |
| T2/T3 | 36 | 50 | 40 |  |
| T4 | 6 | 7 | 7 |  |
| Lymphatic metastasis |  |  |  | 0.348 |
| Yes | 17 | 23 | 25 |  |
| No | 25 | 34 | 22 |  |
| Bone metastasis |  |  |  | 0.492 |
| ＜3 | 22 | 23 | 21 |  |
| ≥3 | 20 | 34 | 26 |  |
| Visceral metastasis |  |  |  | 0.236 |
| Yes | 14 | 11 | 10 |  |
| No | 28 | 46 | 37 |  |
| Gleason score |  |  |  | 0.542 |
| ≤7 | 10 | 10 | 7 |  |
| ＞7 | 32 | 47 | 40 |  |
| ECOG score |  |  |  | 0.882 |
| 0 | 7 | 12 | 7 |  |
| 1 | 23 | 32 | 29 |  |
| ≥2 | 12 | 13 | 11 |  |
| Hypertension |  |  |  | 0.786 |
| Yes | 15 | 23 | 16 |  |
| No | 27 | 34 | 31 |  |
| Diabetes |  |  |  | 0.374 |
| Yes | 12 | 22 | 20 |  |
| No | 30 | 35 | 27 |  |
| Risk |  |  |  | 0.458 |
| High | 22 | 23 | 23 |  |
| Low | 20 | 34 | 24 |  |

**Table 2** Comparison of Disease Progression and PSA Response Among the Three Groups of High-Risk Patients

| Observational parameters | A (n=22) | B (n=23) | C (n=23) | p_value |
| --- | --- | --- | --- | --- |
| Disease progression (Yes, n) | 6(27.3%) | 9(39.1%) | 16(69.6%) | 0.013 |
| PSA response (Yes, n) |  |  |  |  |
| nPSA | 12(54.5%) | 12(52.2%) | 5(21.7%) | 0.044 |
| PSA90 | 14(63.6%) | 13(56.5%) | 6(26.1%) | 0.027 |
| PSA50 | 18(81.8%) | 17(73.9%) | 9(39.1%) | 0.006 |
| PSA progression (Yes, n) | 5(22.7%) | 9(39.1%) | 14(60.9%) | 0.033 |
| Radiographic progression (Yes, n) | 3(13.6%) | 4(17.4%) | 7(30.4%) | 0.340 |

**Table 3** Comparison of Disease Progression and PSA Response Among the Three Groups of Low-Risk Patients

| Observational parameters | A (n=20) | B (n=34) | C (n=24) | p_value |
| --- | --- | --- | --- | --- |
| Disease progression (Yes, n) | 3(15%) | 5(14.7%) | 13(54.2%) | 0.001 |
| PSA response (Yes, n) | |  |  |  |
| nPSA | 13(65%) | 21(61.8%) | 10(41.7%) | 0.210 |
| PSA90 | 15(75%) | 27(79.4%) | 10(41.7%) | 0.007 |
| PSA50 | 18(90%) | 30(88.2%) | 16(66.7%) | 0.061 |
| PSA progression (Yes, n) | 2(10%) | 4(11.8%) | 13(54.2%) | ＜0.001 |
| Radiographic progression (Yes, n) | 1(5%) | 2(5.9%) | 5(20.8%) | 0.121 |


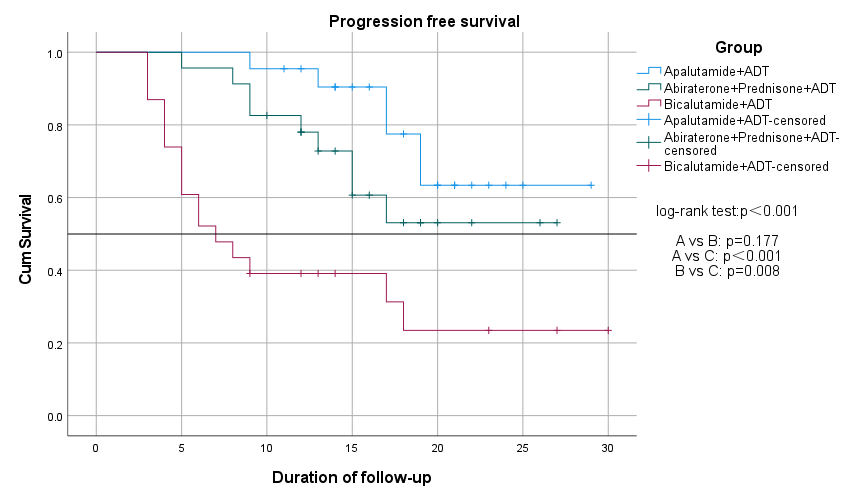


**Figure 1:** Kaplan-Meier Survival Curve for PFS in the High-Risk Group.
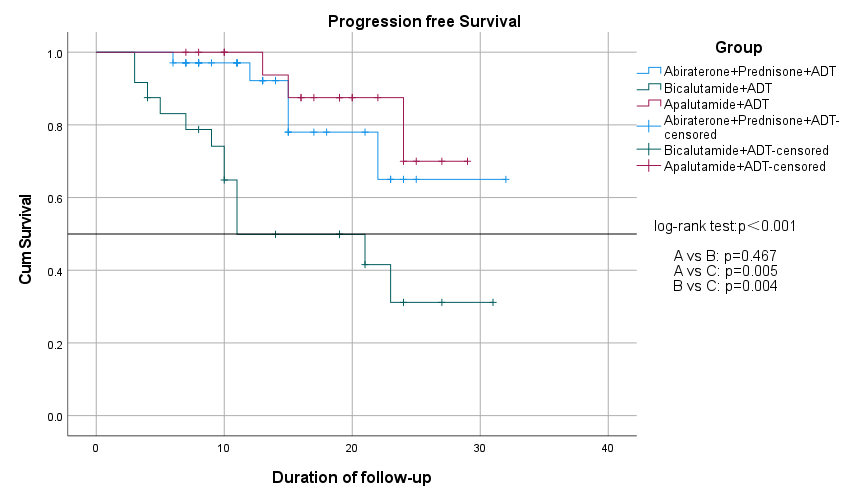


**Figure 2:** Kaplan-Meier Survival Curve for PFS in the Low-Risk Group


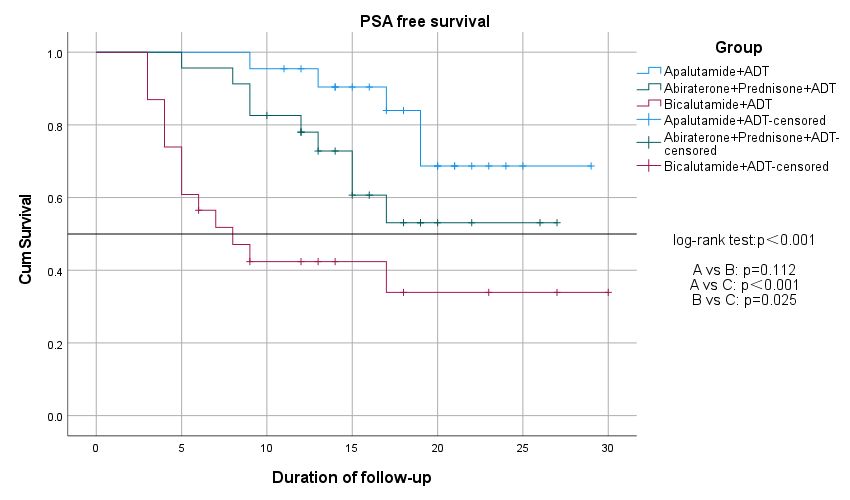


**Figure 3:** Kaplan-Meier Survival Curve for PSA-PFS in the High-Risk Group


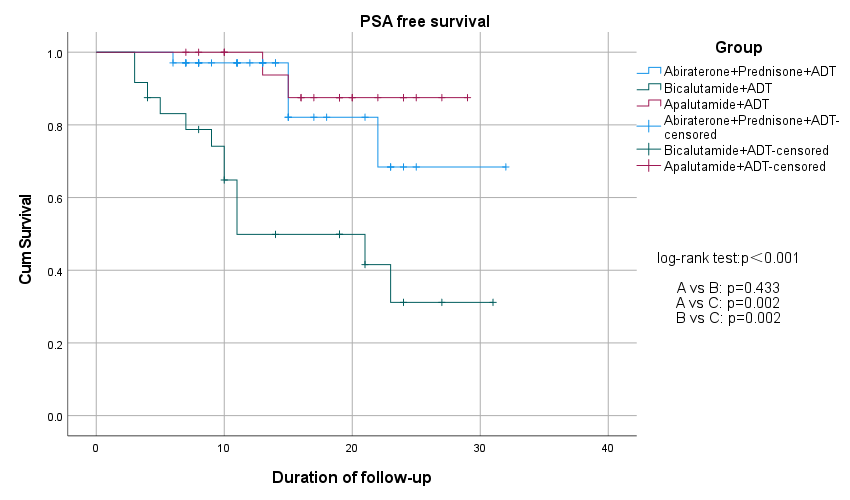


**Figure 4:** Kaplan-Meier Survival Curve for PSA-PFS in the Low-Risk Group

**Table 4:** Occurrence of Adverse Events in the Three Patient Groups

|  | A(n=42) | | | B(n=57) | | | C(n=47) | |
| --- | --- | --- | --- | --- | --- | --- | --- | --- |
| Adverse Events | | Total | Grade ≥3 | | Total | Grade ≥3 | Total | Grade ≥3 |
| Hot flashes | | 6 | 2 | | 2 | 0 | 3 | 0 |
| Rash | | 4 | 0 | | 3 | 0 | 1 | 0 |
| Edema | | 2 | 1 | | 7 | 2 | 3 | 0 |
| Pain | | 10 | 4 | | 8 | 3 | 8 | 4 |
| Fatigue | | 7 | 1 | | 6 | 2 | 4 | 1 |
| Hypokalemia | | 0 | 0 | | 5 | 2 | 0 | 0 |
| Hyperglycemia | | 0 | 0 | | 4 | 0 | 1 | 0 |
| Hypertension | | 3 | 0 | | 2 | 0 | 0 | 0 |
| Gastrointestinal reactions | | 3 | 1 | | 3 | 0 | 11 | 3 |
| Urinary system abnormalities | | 2 | 2 | | 3 | 3 | 4 | 3 |
| Sleep disturbances | | 4 | 0 | | 5 | 0 | 7 | 2 |
| Weight changes | | 3 | 0 | | 4 | 0 | 3 | 0 |
| Abnormal liver and kidney function | | 1 | 0 | | 3 | 0 | 4 | 0 |
| Total number of patients (n) | | 25 | 8 | | 26 | 10 | 23 | 5 |
